# OGO: an ontological approach for integrating knowledge about orthology

**DOI:** 10.1186/1471-2105-10-S10-S13

**Published:** 2009-10-01

**Authors:** Jose Antonio Miñarro-Gimenez, Marisa Madrid, Jesualdo Tomas Fernandez-Breis

**Affiliations:** 1 Departamento de Informática y Sistemas, Universidad de Murcia, Murcia, 30100 Spain; 2 Cell Division Group, Paterson Institute for Cancer Research, University of Manchester, Manchester, M204BX UK

**Keywords:** Gene Ontology, Orthologous Gene, Domain Ontology, Mapping Rule, Gene Ontology Term

## Abstract

**Background:**

There exist several information resources about orthology of genes and proteins, and there are also systems for querying those resources in an integrated way. However, caveats with current approaches include lack of integration, since results are shown sequentially by resource, meaning that there is redundant information and the users are required to combine the results obtained manually.

**Results:**

In this paper we have applied the Ontological Gene Orthology approach, which makes use of a domain ontology to integrate the information output from selected orthology resources. The integrated information is stored in a knowledge base, which can be queried through semantic languages. A friendly user interface has been developed to facilitate the search; consequently, users do not need to have knowledge on ontologies or ontological languages to obtain the relevant information.

**Conclusion:**

The development and application of our approach allows users to retrieve integrated results when querying orthology information, providing a gene product-oriented output instead of a traditional information resource-oriented one. Besides this benefit for users, it also allows a better exploitation and management of orthology information and knowledge.

## Background

Traditionally, biological resources have been designed to be accessed and processed by humans. In such resources, data have been usually been represented in a non-standard format; consequently, data could not be managed and processed appropriately by computers. Hence, the representation of the information in a computer processable manner has become a major research issue. In order to achieve this goal, the availability of computational methods for organizing, accessing and retrieving information in a systematic way has become crucial, as well as the development of methods that allow the definition and maintenance of shared domain models [1].

A large number of biological databases have been developed in the last years. The 2009 update of the Molecular Biology Database collection reveals the existence of more than 1100 databases [2]. There are databases for almost any biological field of study, although most of them contain information about genes and proteins from different organisms. Some examples are the Saccharomyces Genome Database, the Mouse Genome Informatics, Flybase or Wormbase. Most of the initial development efforts were done by reduced research communities, which defined their own terminology. One major limitation of such approach was that research results could not be efficiently used by and shared with other communities. Consequently, new databases were designed to integrate that disperse information in order to provide a common reference for genes and proteins, such as NCBI Entrez [3] or UniProt [4]. Due to the terminological heterogeneity, different groups worked together to develop common vocabularies. That was the origin of the Gene Ontology (GO) [5], which solves the semantic heterogeneity associated to the annotation of gene products between different databases. An ontology is a formal, explicit specification of a shared conceptualisation [6], which provides a shared vocabulary and can be used as a domain model. The success of GO provoked a huge interest in designing, developing and using biological ontologies, whose number has rapidly increased [7]. Projects such as the OBO Foundry [8] promote the development and use of bio-ontologies.

From the technical perspective, ontologies are the cornerstone technology for the Semantic Web [9], which is an extension of the current World Wide Web in which the semantics of web information and services is well defined; hence the web content become understandable by both humans and machines. In fact, different Semantic Web technologies such as RDF [10], OWL [11] and SPARQL [12] have been used for developing semantic biological solutions (see for instance the Semantic Systems Biology portal [13]). In this work, Semantic Web technologies will be used to integrate biological information about orthology.

As stated in [14], it is common practice in biology to obtain key information about the function and evolution of a protein of interest through the identification of homologous proteins in other organisms. There are three types of relevant homologous relations: (1) orthology, which is the relationship between two genes in different species that have evolved from a common ancestral gene via speciation; (2) paralogy, which describes the relationship between two genes concerned to a gene duplication event; and (3) xenology, which describes the relationship between two genes in cases in which one has been derived by horizontal gene transfer. There exist also systems and databases related to orthology, among which the eukarYotic OrtholoGY system (YOGY) [14] is the most important. YOGY is a web-based resource for retrieving orthologous proteins from ten eukaryotic organisms and one prokaryote: *Homo sapiens*, *Mus musculus*, *Rattus norvegicus*, *Arabidopsis thaliana*, *Dictyostelium discoideum*, *Drosophila melanogaster*, *Caenorhabditis elegans*, *Plasmodium falciparum*, *Escherichia coli*, *Schizosaccharomyces pombe*, and *Saccharomyces cerevisiae*.

YOGY is an application that retrieves information about orthology from five independent resources: KOG [15], Inparanoid [16], Homologene [17], OrthoMCL [18], and a table of curated orthologs between budding yeast and fission yeast. The queries are performed by gene or protein identifier. Moreover, YOGY is able to associate GO terms to the genes included in the search results. The integration of such complementary data provides a practical tool for identifying known or predictable parenthood relations between proteins from different species.

The clusters of orthologous genes are shown by resource in YOGY, thus providing a common, integrated query interface, which implies that searches can be done in all the databases by using one query. However, the results retrieved include redundant information about genes and proteins and, consequently, the benefits of providing a common query interface are minimized by the non integration of the output information. This is a clear drawback for both humans and machines, because it makes the automatic, efficient analysis and processing of such information more difficult. As it has already been mentioned, YOGY searches for biological terms in independent databases, and it shows the results by resource. The meaning of the information contained in each repository is unknown for machines, so YOGY cannot analyze, compare and integrate the data or do any knowledge-intensive task.

Hence, this work aims at providing mechanisms for integrating the information of the resources used by YOGY. The results should not be shown by resource in an independent manner, including redundant items, but in an integrated one, allowing users to know the origin of data. Hence, the exploitation of the information can be semantics-driven, making it processable by machines, facilitating its understanding and increasing its usefulness to researchers.

In the last years, different methods have been applied to data integration in biomedical domains: federated systems, ontology-based mediation systems, data warehouse and workflows [19]. These range from the usage of an individual model or parser per resource to the representation of the information sources in a common format. Other methods are based on the application of RDF, OWL and SPARQL to integrate, store, query, and view the information from different resources. Some examples are the Thea portal [20], Biomart [21, 22] for the integration of phenotypes and genotypes, and Automed [23] for reconciling biomedical services.

However, the use of heterogeneous schemas makes it usually more difficult for users to query the resources in an integrated way. Semantic solutions have been applied to this end. An example is BioMoby [24], which defines an ontology-based messaging standard for the automatic discovery and interaction with biological data and data analysis service providers.

In summary, the integration of biomedical resources is necessary due to the huge amount of information that is continuously being generated and considering its inherent diversity and heterogeneity. Otherwise, the mentioned caveats would limit the efficient use of such resources. In this work, a Semantic Web-based approach for the integration of biological knowledge will be applied to orthology. The integration will be facilitated by a domain ontology, which will be used for mapping the different resources to achieve a common machine processable representation. This will allow the generation of an integrated ontological knowledge base, to which biologists will have access through a user-friendly web interface.

## Results

**Figure 1:**
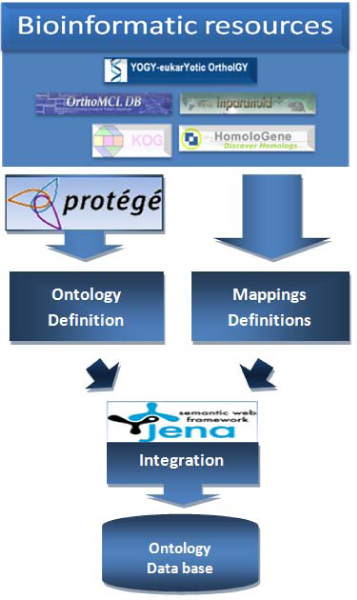
**The Ontological Gene Orthology methodology**. The methodology has four main steps: (1) Information capture from the resources; (2) Design of the domain ontology, in this case, the orthology ontology; (3) Definition of the mapping rules between the resources and the ontology, which aligns the entities used in the resources and the ones defined in the ontology and which will drive the data integration process; and (4) Integration of the information to generate the ontological knowledge base.

The Ontological Gene Orthology (OGO) methodology has been developed (see Figure 1) for integrating, managing and exploiting biological information about orthology. This methodology is based on Semantic Web technologies, which facilitate handling the biological data and orthology-related knowledge and support the development of integration processes using the semantics of the domain. This methodology (see the Methods section for further details) has four steps: (1) capture of the information from the resources; (2) development of the ontology; (3) definition of the mappings between the resources and the ontology; and (4) construction of the integrated ontological knowledge base.

The application of this methodology to orthology resources has generated three main results: (1) an ontology about orthology; (2) an integrated ontological knowledge base about orthology; and (3) a user-friendly web interface for querying the semantic repository.

### The OGO ontology

**Figure 2:**
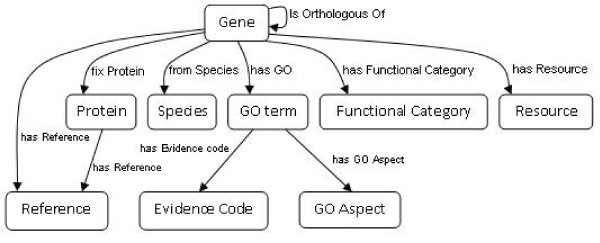
**Partial representation of the OGO ontology**. The central concept is Gene. Each box represents a concept of the ontology and each arrow represents a relation between concepts. This ontology includes the aspects of interest for integrating the target bioinformatics resources.

This ontology has been developed to model the knowledge related to orthology (see Figure 2) and to guide the integration of data by means of mappings between the resources and the concepts of the ontology. It was the result of analyzing the bioinformatics resources used in this work: KOGs, Inparanoid, Homologene, OrthoMCL, NCBI Taxonomy, and Gene Ontology. Since it contains the concepts, relationships and restrictions of the domain, the consistency of the data integrated can also be checked.Next, the most important concepts of the ontology are described:

#### Gene

This is the most important concept and it has many relationships associated. The property *isOrthologousOf* represents the orthologous relationship between genes and it is defined as symmetric. The property *hasReference* connects genes with concepts that define how genes are identified and named in the different resources, so genes may have several references. The property *hasResource* represents the relation between a *gene* and the bioinformatics resource from which the information is extracted. A gene has to be in at least one resource. The concept *species* is related with *gene* through the property *fromSpecies*. *Genes* and *proteins* are linked through the property *fixProtein*. Genes may also have functional categories associated, which are represented by the property *hasFunctionalCategory*, and this information is obtained from KOG. Finally, each *gene* has different *GO terms* associated in the property *hasGO*.

#### Protein

Analogously to genes, proteins have also the property *hasReference* which includes identifiers and names of proteins in different resources.

#### Resource

This concept represents the repositories from which genes and proteins are collected.

#### Species

This is a taxonomy obtained from NCBI species taxonomy database. It has 33 concepts and about 100 organisms.

#### Category

25 functional categories obtained from KOGs have been included in the ontology. These functional categories are grouped into 4 main subsets: cellular processes and signalling, information storage and processing, metabolism and poorly characterized. They are the functions of the orthologous group to which a particular gene belong.

#### GO

GO terms are related to genes but also linked to other concepts in the ontology. The property *hasEvidenceCode* connects GO terms and evidence codes. The ontology contains 6 types of evidence codes: author statement, automatically-assigned, computational analysis, curator statement, experimental and obsolete. This taxonomy has 18 instances. Besides, a GO term is connected to GO aspect. The ontology includes three GO aspects: biological process, cellular component, and molecular function.

The ontology was implemented in the Web Ontology Language (OWL) by using Protégé [25]. OWL is the current W3C recommendation for the exchange of semantic content on the Web and it is the language used by the Semantic Web community for representing ontologies. Our OWL ontology contains 52 concepts, 9 object properties, and 2 data type properties. Cardinality constraints and disjoint axioms have also been defined in the ontology. Figure 2 shows the most relevant concepts (as boxes) and relationships (as arrows) of the OGO ontology.

### The integrated ontological knowledge base

As it has already been mentioned the OGO ontology has been used to guide the integration of the orthology resources. As a result of this process, the OGO knowledge base was obtained, containing approximately 1,168,000 orthologous genes, 956,000 proteins related to those genes, and 114,000 orthologous clusters.

Due to the huge amount of individuals, efficient and scalable methods for building the knowledge base are required. The Jena Semantic Web Framework [26] was used to develop the repository, because it provides relational persistence for ontology models, as well as the use of reasoners and semantic query languages. In this work, the relational persistence was provided by the MySQL open source relational dataset [27], and Pellet [28] was the reasoner used to check the consistency of both the ontology and the integrated instances.

### The web tool

**Figure 3:**
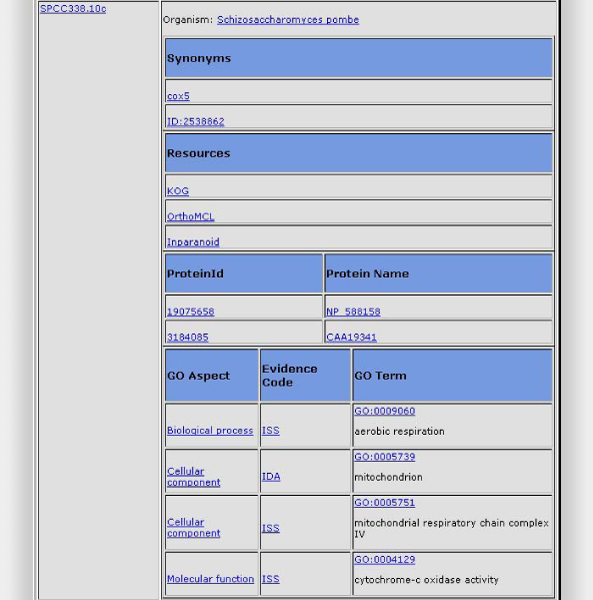
**The OGO query interface**. It allows for querying by gene id or name, as well as filtering the properties to include in the results. This figure is a screenshot of the results obtained by OGO for the gene *cox5*. For this gene, the synonyms, resources from which the information has been retrieved, its associated proteins and its associated GO terms are shown.

The OGO knowledge base was built using semantic technology so that languages such as SPARQL can be used for querying it. Since our goal is to provide access to biologists to integrated information about orthology and, provided that we cannot expect or force them to master languages such as SPARQL, alternative query methods are needed. To solve this problem the web interface shown in Figure 3 has been developed. This interface allows for querying the knowledge base using both the protein or gene identifiers, thus retrieving orthology information about genes, species, information about their corresponding proteins and Gene Ontology terms. Besides, it permits users to filter the information by species and by bioinformatics resource. This tool can be accessed at OGO Portal [29].

Figure 3 is an example of the results returned by the OGO web interface. This figure consists of two columns, the left side one contains the gene identifier and the right side contains the information retrieved for the gene: organism, synonyms, resources, related proteins and Gene Ontology terms. Users can select what information has to be included in the results by using the corresponding filters. Links to the resources are displayed and can be accessed by the users to get more information about the particular result. For instance, the organism is linked to the NCBI taxonomy database, and each synonym is linked to repositories that contain that information about them. Resources are the databases from which the information was collected and integrated for that particular gene. Protein information consists of two columns, protein id and protein name. Finally, the Gene Ontology information consists of three columns: GO Aspect, Evidence Code, and GO term. Each row corresponds to a GO term, these terms being alphabetically sorted by GO Aspect. GO term cells contain the identifier and a brief description. This information is linked to the Gene Ontology browser AMIGO [30].

## Discussion

Traditionally, bioinformatics databases have been represented using flat files. In this work we have approached the representation of data and knowledge using OWL, which allows humans not only to understand the contents but also software tools to use the biological knowledge for different purposes. Furthermore, formal representation technologies and languages such as ontologies and OWL provide native mechanisms for ensuring the consistency, quality and correctness of the contents which cannot be granted by flat text files and relational databases.

This technology facilitates the integration of biological information as well as its connection with other repositories by adding more concepts and relationships into the ontology. In fact, we are currently defining the mappings between our ontology and relevant bio-ontologies to achieve this objective.

It should be noted that the data captured from the resources is not integrated on the fly. The files of the resources are periodically updated by their developers, so they are periodically downloaded and the knowledge base construction process is launched. We have tried to make this process automatic, but this has not been possible to date, mainly because of problems caused by the structure of the data files. The files available for downloading are not always well-formed and are not precise enough for enabling automatic machine processing. Therefore, some manual pre-processing is needed to identify and repair the structural errors of the files according to their specifications. Then, the automatic process for information retrieval and integration is launched and the knowledge base is updated.

Another advantage of our approach is that the ontological knowledge base allows queries with semantic languages such as SPARQL. Thus, we can incorporate in the queries all the restrictions modeled from the biological domain and represented in the ontology. The current web interface allows for a reduced set of queries, since our initial goal is to provide easy access to integrated information about orthology. We plan to extend such interface to allow users to make more complex semantic queries in a friendly way.

The response time of the system has also been considered. The performance of the ontological repository is worse than using a relational database, although our experiments with more than 100 simultaneous, simulated users have not reported significant problems, so the new possibilities by the semantic technology are worthwhile.

The OGO methodology has been applied in this work, and it could easily be adapted to cover new orthology resources. It might also be adapted to other biological areas in which data integration is required.

Finally, YOGY was a reference system for us, since we shared the same biological goals but used a different technological approach. We think there are three main aspects in which the OGO approach outperforms YOGY:

1. Less redundant information is retrieved in OGO because of data integration. YOGY retrieves information from each resource and this is displayed to the user on a resource-by-resource basis, so the users have to manually combine the results. OGO performs an integration process, which makes the exploration of the results easier for the users.
2. Our repository integrates all the information available from the biological resources, whereas YOGY is specialized in ten eukaryotic organisms and one prokaryotic.
3. The existence of inconsistencies can be controlled by the appropriate use of reasoners in OGO, so that inconsistent results can be removed from the set of results. This cannot be done in YOGY due to the non-integration of data.

## Conclusion

In this paper, we have presented the OGO approach for integrating and managing bioinformatics resources that contain information about orthology. First, we analyzed the resources to develop the domain ontology, which supported the integration of data. As a result of this process, the ontological knowledge base was obtained, and the friendly user interface was developed. The results show the potential of Semantic Web technologies to represent biological knowledge, to facilitate the integrated access to biological data and to support the development of semantics-rich applications for biologists. The semantics of the domain has been used in this work to support the integration and the query of the knowledge base, showing its advantages against traditional systems.

## Methods

This work has been focused on integrating the information resources included in YOGY to build an integrated ontological knowledge base. The OGO methodology was shown in Figure 1, consisting on four main steps:

1. Capturing information from the resources.
2. Designing the orthology ontology.
3. Defining mappings between the resources and the ontology, which are needed to support data integration processes.
4. Integrating information into the ontological knowledge base.

### Capturing information from resources

This is the first step of the methodology, whose objective is to process the information sources to get the uniform representation of data. In this work, KOGs, Inparanoid, Homologene and OrthoMCL have been the resources used. The files of each resource used in this work are shown in Table 1.

**Table 1:** Resource files used in this work. This table describes the resource files which contain the orthology information used in this work. For each resource, the name of the files used, their size and version is displayed. These files contain the data that has been integrated in OGO. The file structure of each resource is different. For instance, Homologene has only one file, whereas Inparanoid has 595. Hence, different processing mechanisms are needed for each resource.

Resource	File	Size (in KB)	Version
KOG	kyva=gb.txt	2098	06/06/2003
KOG	kog.txt	1312	21/07/2003
Inparanoid	595 files in tables_stats directory	1153434	22/04/2008
OrthoMCL	all_orthomcl.out	8631	20/07/2006
OrthoMCL	BAE_geneid_anno.txt	40913	20/07/2006
Homologene	homologene.data	10846	28/07/2008

Next, the data captured from each resource is described:

#### KOG

The euKaryotic Orthologous Groups of proteins database (KOG) is part of the Cluster of Orthologous Groups (COG) developed by the NCBI. In particular, the files *kog.txt* and *kyva=gb.txt* have been used in this work. The file *kog.txt* contains the orthologous cluster references, their own functional categories and the gene identifiers. The file *kyva=gb.txt* contains a list of two columns of related genes and proteins.

#### Inparanoid

This resource has information about clusters of orthologous genes and proteins of 35 organisms, organized in 595 files. Each file contains the genes and proteins of an organism that are orthologous to the genes and proteins of another.

#### Homologene

It is part of the NCBI databases, which contains information about clusters of orthologous genes. The information from this resource was obtained from the file *homologene.data*, and it consists of six columns: orthologous cluster identifier, species identifier, gene identifier, gene symbol, protein GenBank identifier, and protein accession number.

#### OrthoMCL

It contains information about orthologous genes from 87 organisms. This information is classified in groups of orthologous proteins. The file *all_orthomcl.out* contains information about orthologous clusters, and the file *BAE_geneid_anno.txt* connects genes and proteins through the accession numbers used in the OrthoMCL database.

Since the information of these resources is limited to orthology, complementary information was gathered from other bioinformatics resources. This information permits to connect genes and proteins instances among resources. The information was basically collected from NCBI databases of genes, proteins, Gene Ontology terms and species. Other biological information such as alternative gene and protein names were retrieved from Ensembl and Uniprot, respectively.

### Designing the ontology

The OGO ontology (see Figure 2) represents the knowledge of the orthology domain managed by the bioinformatics resources. The knowledge base uses this conceptualization for storing and navigating information in the repository. The ontology was developed by analyzing each resource independently. The result was a partial ontology for each resource. Then, an integrative approach, based on the one proposed in [31], was used to generate the global OGO ontology. Finally, the ontology was written in OWL. This approach for building the ontology also facilitated the definition of the mappings between resources and the OGO ontology.

### The mappings

The mappings between the resources and the ontology drive the integration process, since they define the correspondence between the particular viewpoint of a resource and the global one of the OGO ontology. The inputs of this step are the data files included in Table 1, the partial ontologies and the OGO ontology. The result of this phase is a set of mapping rules that will be used for inserting the information in the ontological knowledge base. Next, the mapping rules defined for each resource are described.

#### KOG mapping rules

**Figure 4:**
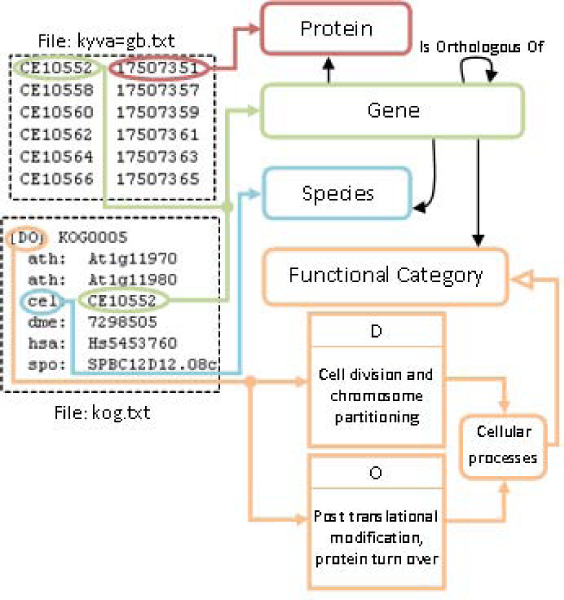
**Graphical representation of an example of mapping rules for KOG**. The information in KOG is divided in two files: *kog.txt* and *kyva=gb.txt*. A mapping rule was defined for connecting orthology cluster information from *kog.txt* to gene and protein information from *kyva=gb.txt*. On the other hand, the other mapping rules link this orthology information to the OGO ontology. So *cel:CE10552* from *kog.txt* is connected with *CE10552 17507351* from *kyva=gb.txt* and also the fields of both files are mapped to the corresponding concepts in the OGO ontology.

The relevant information was obtained from the files *kog.txt* and *kyva=gb.txt*. The first one contains clusters of orthologous genes, with their functional categories and species associated, whereas the second one contains the relations between genes and proteins. Figure 4 is an example of mapping rules applied to a cluster of orthologous genes from *kog.txt* and *kyva=gb.txt*. In *kog.txt*, the functional categories appear in the first line between square brackets, and each character represents one functional category. The names of the functional categories and their corresponding codes were gathered from KOG. Instances of each functional category were created with their identifier as a label in the ontology, hence clusters only have to reference them.

The file follows with the representation of the orthologous genes of the cluster; the species identifiers, which are a three characters string, and the gene identifiers. The list of species used in the resource and its label identifiers were extracted from KOG and instantiated in the ontology. Each individual has then a label with its identifier.

In *kyva=gb.txt*, gene identifiers appear in the left column, whereas NCBI protein identifiers appear in the right one. There is a correspondence between these gene identifiers and the ones in *kog.txt*, so this information can be merged.

The mapping example shown in Figure 4 corresponds to the gene *CE10522* from *Caenorhabditis elegans*, which is connected to the protein with identifier *7507351*. This gene instance will be related to other genes instances by means of the property *isOrthologousOf*. Finally, since the species and functional categories were manually added to the ontology, we defined mapping rules that connects them with the corresponding instances in the ontology.

#### Inparanoid mapping rules

This resource consists of 595 files about 35 organisms and each file contains information about orthology from two different species. For example, the file *InParanoid.ensBOSTA-ensRATNO* has the orthologous clusters of *Bos taurus* (BOSTA) and *Rattus norvegicus* (RATNO).

**Figure 5:**
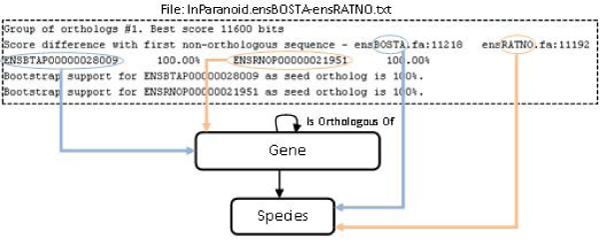
**Graphical representation of an example of mapping rules for Inparanoid**. This resource consists of 595 files with orthology information from a pair of organisms. A mapping rule allows for gathering all files in a single one in order to obtain the orthology clusters. Then, mapping rules such as the ones represented in this figure connect the orthology information to the corresponding concepts in the OGO ontology. We can see in this figure how the species and genes identifiers are associated to the corresponding concepts of the ontology.

The example shown in Figure 5 has two genes from *Bos taurus* and *Rattus norvegicus*. The third line allows to obtain both identifiers and their orthologous relations. Provided that some groups do not have a complete orthologous relation, a confidence value of 100% of accuracy has been applied to obtain orthologous genes.

In order to create the orthologous clusters which integrate all the organism information, a mapping rule that connects the records of common organisms was defined, so a pre-integration step is here performed. As a result of this process, a single file is obtained, which simplifies the integration process. Another rule was defined for mapping gene identifiers in the file to gene instances in the ontology.

#### OrthoMCL mapping rules

The file *all_orthomcl.out* contains the information about orthologous clusters. There, each line represents a list of orthologous genes that contains private gene identifiers and their species identifiers. On the other hand, the file *BAE_geneId_anno.txt* contains two columns that connect the private gene identifiers with their public ones.

**Figure 6:**
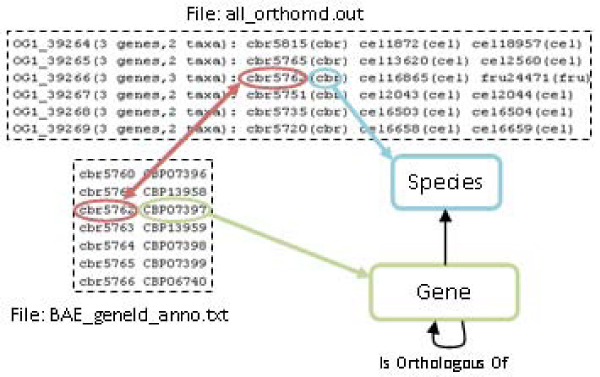
**Graphical representation of an example of mapping rules for OrthoMCL**. The *all_orthomcl.out* file contains the orthology cluster information, and *BAE_geneId_anno.txt* contains the gene names that appear in the previous file. As can it be noticed, this resource uses private identifiers for storing and linking information across files. Therefore, the rule uses this identifier for establishing the mapping between both files. There are also other mapping rules to link the orthology information to the concepts in the OGO ontology. So, *cbr5762(cbr)* from *all_orthomcl.out* is connected with *cbr5762 CBP07397* from *BAE_geneId_anno.txt* and the fields of both files are also mapped to the corresponding ontology concepts.

These mapping rules are described in Figure 6. The private identifiers are used to connect the orthologous clusters with public gene identifiers. Another mapping rule associates the identifiers with their corresponding instances in the ontology.

#### Homologene

**Figure 7:**
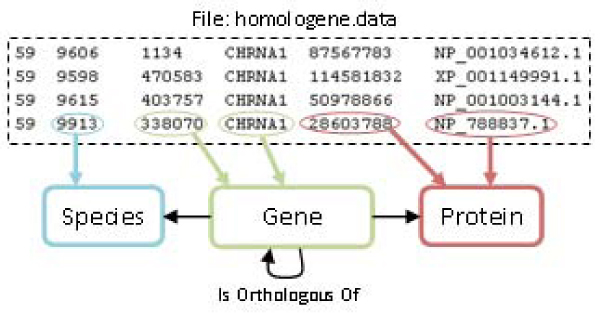
**Graphical representation of an example of mapping rules for Homologene**. This resource consists of a single file that contains all the orthology information available. This file consists of six columns: (1) cluster identifier; (2) organism identifier; (3) gene identifier; (4) gene name; (5) protein identifier; and (6) protein name. The mapping rules shown in the figure link each column data to the corresponding concepts in the OGO ontology.

The file *homologene.data* contains clusters of orthologous genes of many species. Figure 7 shows an extract of the file which has six columns: (1) cluster identifier; (2) species identifier; (3) gene identifier; (4) gene symbol; (5) protein GenBank identifier; and (6) protein accession number. The definition of the mappings required a previous collection of information from other databases which use the same identifiers and tags for referencing gene instances already existing in the ontology. This resource uses NCBI taxonomy for identifying the organism. Therefore, we retrieved such information and combined it with the ontology instances through a mapping rule.

#### Other mapping rules

**Figure 8:**
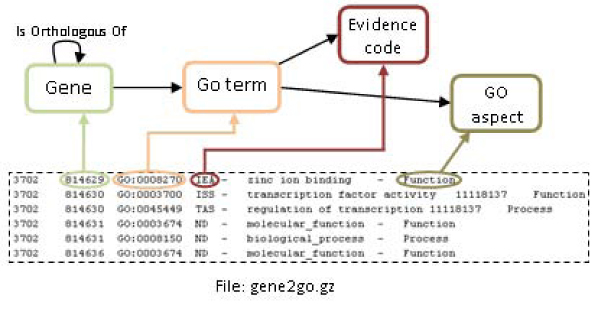
**Graphical representation of an example of mapping rules for Gene**. This figure shows the mapping rules defined for linking the information contained in gene2go.gz file. This file was collected from NCBI and contains references to gene identifiers, Gene Ontology terms, aspects and evidence codes. This information was collected in order to enrich the biological information stored in the system.

Due to the lack of information about genes and proteins in some resources, biological information was gathered from additional ones. The most important of them was NCBI Gene, which permits the retrieval of gene information from the files *gene_info.gz* and *gene2accession.gz*. These files contain useful data to complete the orthologous genes and proteins information. In this way, information about proteins and their related genes was added to the knowledge repository. This information facilitates the integration since genes and proteins identifiers are important agents in the integration process. Besides, another file was collected from NCBI, *gene2go.gz*. This file contains references to Gene Ontology terms, aspects, evidence codes, and their gene identifiers and how these parts are mapped onto the ontology is described in Figure 8.

The species taxonomy was collected from the NCBI Taxonomy database. This was necessary because some resources use taxonomy names and identifiers. Although most of the information was collected from NCBI databases other resources like Ensembl and Uniprot were needed to provide alternative gene and protein names, which were useful for mapping instances to the knowledge repository.

### Information integration

Once the mapping rules between the resources and the OGO ontology have been defined, the information can be integrated. This process has three main steps: (1) application of the intra-resource rules; (2) information enrichment; and (3) sequential execution of the resource-to-ontology mapping rules.

The first step executes the mapping rules that link information from the same resource, for instance, those relating *kog.txt* and *kyva=gb.txt* from KOG. This is necessary when the information resource has more than one data file. Intra-resource rules have been defined for all the resources except for Homologene. As a result, we obtained an integrated representation of each resource. The second step allows for adding information from NCBI, Ensembl and Uniprot to the orthologous gene and protein instances of each individual resource. Finally, the third step generated individuals of the OGO ontology from the gene and protein instances of each individual resource by applying the mapping rules between each resource and the ontology.

Hence, the proper semantic integration is performed in the third step and is basically guided by the gene and protein properties used in the resources. The comparison of two instances is done in terms of their properties and identifiers. If an instance is a subset of the other instance, then the information of their orthologous clusters is merged to avoid redundancy. In fact, two individuals are not allowed to have the same name and properties, so redundancy can be controlled. The definition of the OGO ontology also included restrictions to avoid inconsistencies. The restrictions defined in this ontology were disjointness, allValuesFrom (to avoid inconsistencies in the range of object properties); and minCardinality and maxCardinality (to control the cardinality of properties). The Jena Semantic Web Framework is capable of detecting such issues so it facilitates the verification of consistency when used together with reasoners such as Pellet.
